# Time-Based Partitioning Model for Predicting Neurologically Favorable Outcome among Adults with Witnessed Bystander Out-of-Hospital CPA

**DOI:** 10.1371/journal.pone.0028581

**Published:** 2011-12-14

**Authors:** Toshikazu Abe, Yasuharu Tokuda, E. Francis Cook

**Affiliations:** 1 Department of Emergency Medicine, Mito Kyodo General Hospital, University of Tsukuba, Mito City, Ibaraki, Japan; 2 Institute of Clinical Medicine, Graduate School of Comprehensive Human Sciences, University of Tsukuba, Mito City, Ibaraki, Japan; 3 Department of Epidemiology, Harvard School of Public Health, Boston, Massachusetts, United States of America; Virginia Commonwealth University, United States of America

## Abstract

**Background:**

Optimal acceptable time intervals from collapse to bystander cardiopulmonary resuscitation (CPR) for neurologically favorable outcome among adults with witnessed out-of-hospital cardiopulmonary arrest (CPA) have been unclear. Our aim was to assess the optimal acceptable thresholds of the time intervals of CPR for neurologically favorable outcome and survival using a recursive partitioning model.

**Methods and Findings:**

From January 1, 2005 through December 31, 2009, we conducted a prospective population-based observational study across Japan involving consecutive out-of-hospital CPA patients (N = 69,648) who received a witnessed bystander CPR. Of 69,648 patients, 34,605 were assigned to the derivation data set and 35,043 to the validation data set. Time factors associated with better outcomes: the better outcomes were survival and neurologically favorable outcome at one month, defined as category one (good cerebral performance) or two (moderate cerebral disability) of the cerebral performance categories. Based on the recursive partitioning model from the derivation dataset (n = 34,605) to predict the neurologically favorable outcome at one month, 5 min threshold was the acceptable time interval from collapse to CPR initiation; 11 min from collapse to ambulance arrival; 18 min from collapse to return of spontaneous circulation (ROSC); and 19 min from collapse to hospital arrival. Among the validation dataset (n = 35,043), 209/2,292 (9.1%) in all patients with the acceptable time intervals and 1,388/2,706 (52.1%) in the subgroup with the acceptable time intervals and pre-hospital ROSC showed neurologically favorable outcome.

**Conclusions:**

Initiation of CPR should be within 5 min for obtaining neurologically favorable outcome among adults with witnessed out-of-hospital CPA. Patients with the acceptable time intervals of bystander CPR and pre-hospital ROSC within 18 min could have 50% chance of neurologically favorable outcome.

## Introduction

The critical lifesaving steps of Basic Life Support (BLS) include immediate recognition, activation of the emergency response system, and early cardiopulmonary resuscitation (CPR), immediate defibrillation for ventricular tachycardia/ventricular fibrillation (VT/VF) [1]. When an adult suddenly collapses, whoever is nearby and witnesses it should activate the emergency system and begin chest compressions regardless of his/her experience for CPR training. However, some laypersons could not provide bystander CPR. They might hesitate to provide CPR or start CPR after dispatch assistants come to the scenes. Traditionally, one minute delay of CPR initiation may result in about 10% reduction of survival chance [2]. Thus, 10-minute delay of CPR initiation might indicate there are few chances for CPA patients to survive.

It may also be more important to ensure shorter time intervals from the time of patient collapse to the crucial points of CPR course than the choices of treatment modalities and procedures especially in pre-hospital CPR settings. Uninterrupted continuation of CPR is crucial for survival or neurologically favorable outcome and prolonged interruptions in chest compressions should be avoided during basic and advanced life support. Studies indicated that automated external defibrillator (AED) use in hospital was associated with poor outcomes and that this reason might be related to greater interrupted time of CPR in those with AED use [3], [4], [5]. Although dispatcher assistance is recommended, it did not always lead to better outcome because it often makes CPR delay [6]. Likewise, the increased rates of ROSC associated with Advanced cardiac life support (ACLS) drug therapy have yet to be translated into long-term survival benefits [7]. Presumably, it would take a long time on the scene to use epinephrine, intravenous fluid (IV), airway tools by emergency medical service (EMS) or dispatch assistances than simple transportation with BLS because of a limited number of staffing at pre-hospital settings.

However, it has been unclear about optimal recommended time interval to CPR initiation from collapse of CPA patients for survival or neurologically favorable outcome. Few studies were available for focusing on thresholds about acceptable time interval from the time of collapse to crucial points of CPR course for survival or neurologically favorable outcome. Previous studies might not have controlled time factors effectively because there was little evidence about the acceptable time interval of CPR course. Therefore, in this study, our purpose was to assess optimal recommended time interval to CPR initiation from collapse of CPA patients for one-month survival or neurologically favorable outcome one month after admission and to develop a prediction model for neurologically favorable outcome and survival among CPA patients.

## Methods

### Ethics Statement

The ethics committee at our institution does not require its approval for observational studies using anonymous data in existence such as this study. Also, informed consent from each patient was waived for using anonymous data according to the informed consent guidelines in Japan [8]. We obtained these anonymous data with the permission from the Fire and Disaster Management Agency (FDMA) [9] in the Ministry of Internal Affairs and Communications. We had been conducted this research according to the principles expressed in the Declaration of Helsinki.

### Study design and data collection

In Japan, calls to the universal emergency access number 119 are directly connected to a dispatch center located in the regional fire defense headquarters, covering 807 fire stations as of 2007. On receipt of a call, the nearest available ambulance is sent to a scene [10]. This comprehensive emergency network with the EMS covers the entire Japan, which is administered by the FDMA.

Our study employed nationwide population based prospective observational design, which involved consecutive patients with out-of-hospital CPA in Japan from 2005 to 2009. We collected 547,218 cases on the nationwide database developed by the FDMA. All patients in this database experienced CPA outside medical facilities and were transferred to hospitals. They were classified as CPA confirmed by EMS on arrival at the scene, CPA in an ambulance during transfer to hospital, or supposed CPA in which the patient had already been resuscitated when the EMS arrived. The database involved all cases of out of hospital CPA in which patients was transported to hospital because it is only one nationwide system of ambulance service in Japan.

The data on out-of-hospital CPA were collected by the EMS in the local fire departments from their observation and interviews with bystanders and physicians in charge of the patients with out-of-hospital CPA. Data were obtained on age, sex, whether the collapse was witnessed, whether bystander CPR was performed, conventional CPR (or chest-compression only CPR), AED used by a bystander, whether the dispatcher gave assistance by telephone, the category of bystander (such as lay person or EMS), initial identified cardiac rhythm, DC (Defibrillator Cardioversion) used by EMS, kinds of defibrillator, category of airway tools by EMS, IV used by EMS, epinephrine used by EMS, and cause of cardiac arrest such as cardiac or non-cardiac origin. We also obtained data for time intervals from collapse to bystander CPR course including the interval from collapse to CPR initiation, the interval from collapse to ambulance arrival, the interval from collapse to hospital arrival, and the interval from collapse to ROSC at pre-hospital. EMS also obtained outcome data from physicians in charge about survival and category of cerebral performance [11] one month after hospital admissions. Physicians in charge, in collaboration with emergency medical service staff, clinically determined the cause of cardiac arrest. EMS also interviewed bystanders on site to determine the length of time from collapse to the first resuscitation attempt and identified the initial cardiac rhythm.

EMS entered all information at local fire departments online, which basically conformed to the Utstein [12] form with some additions. The data were verified by EMS and transferred and stored on the database at the FDMA. The database was verified by the computer system and compiled for public use by the FDMA.

### Selection of Participants

**Figure 1:**
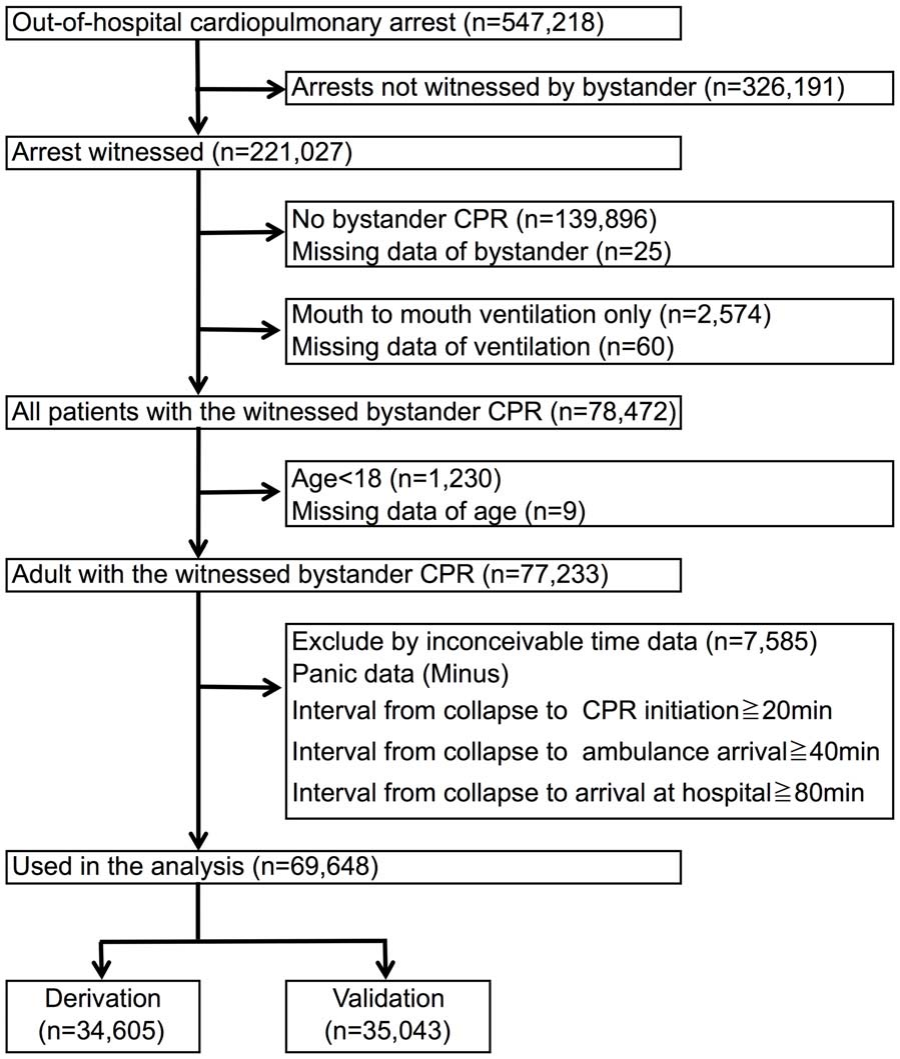
Study profile with selection of participants.

A total of 547,218 subjects were enrolled in the database from 2005 to 2009. Of these, 326,191 patients were excluded because their arrests were not witnessed by bystanders. We also excluded 139,896 patients who did not receive bystander CPR. Further, we excluded 2,574 patients who received only mouth-to-mouth ventilation and 1,230 patients with 17 years of age or younger. Additional 94 were excluded because of missing data. Thus, 77,233 patients met our inclusion criteria. Then, we excluded 7,585 patients with inconceivable time data: the interval from collapse to CPR initiation was minus or greater than 20 min; the interval from collapse to ambulance arrival was minus or greater than 40 min; the interval from collapse to arrival at hospital was minus or greater than 80 min. These data were considered as panic data because of inaccuracy of their recalls or because CPR should be terminated at pre-hospital. Finally, we used data of 69,648 (90.2%) patients for analysis. (Figure 1)

**Figure 2:**
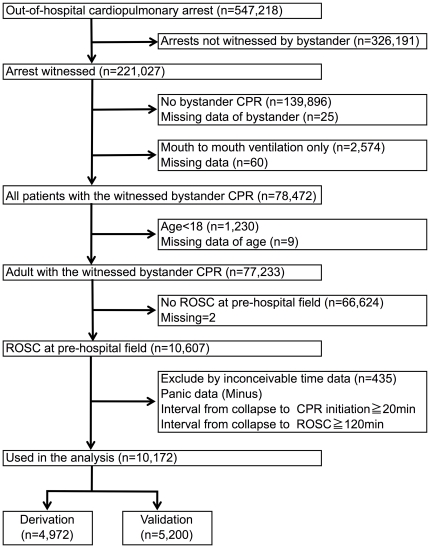
Study profile with selection of participants of the subgroup analysis.

We randomly divided this cohort into two parts, a derivation and a validation dataset for developing a prediction rule. Of 69,648 patients, 34,605 were assigned to the derivation data set and 35,043 to the validation data set. (Figure 1) As a subgroup analysis, we chose 10,607 patients who had a return of spontaneous circulation (ROSC) at pre-hospital. And we used 10,172 (95.9%) patients with conceivable time data for the analysis. Of 10,172 patients, 4,972 were assigned to the training data set and 5,200 to the testing data set. (Figure 2)

### Statistical Analysis

Variables of patient characteristics and outcomes were analyzed as mean ± SD or counts (percentages). Time data were analyzed using median ± interquartile (IQ) because of the non-normal distribution. Primary outcome of our study was proportions of patients with neurologically favorable outcome one month after admissions and secondary outcome was one-month survival. Neurologically favorable outcome was defined as category one (good cerebral performance) or two (moderate cerebral disability) of the cerebral performance categories [11]. Physicians in charge recorded data for survival and category of cerebral performance one month after hospital admissions. EMS obtained those outcome data from them.

By using the derivation dataset, a recursive partitioning model was fit by analyzing the relationship between proportions of neurologically favorable outcome or survival one month after admission and the optimal recommended thresholds of the time intervals of CPR. First, the recursive partitioning was used to identify the optimal time point about data of the interval from collapse to CPR initiation for serially splitting patients (parent nodes) into those with two more homogeneous groups (daughter nodes) in terms of study outcomes. Next, the similar procedure was used to identify the optimal time point about data of the interval from collapse to ambulance arrival and the optimal time point about data of the interval from collapse to hospital arrival. The recursive partitioning was conducted by using maximizing the Entropy index [13]. The similar process for developing a partitioning model in another training dataset was conducted for our subgroup analysis. For the validation of the model, we analyzed the performance accurately predicting proportions of neurologically favorable outcome or survival by using the validation dataset of the primary analysis and testing dataset of the subgroup analysis. JMP version 9.0 (SAS Institute, Cary, NC) was employed for the recursive partitioning analysis and the other analyses were performed with SAS statistical software version 9.2 (SAS Institute, Cary, NC).

## Results

Characteristics of patients, bystanders CPR, EMS, and the time intervals of CPR (the derivation and validation dataset combined) are shown in Table 1. Mean age was 74.6±15.8 yrs and 57.8% of patients were men. The median (25%–75% interquartile) intervals from collapse to important events in all patients (N = 69,648) were: 1 (0–3) min from collapse to CPR initiation; 9 (7–12) min from collapse to ambulance arrival; and 32 (26–41) min from collapse to hospital arrival. In the subgroup with pre-hospital ROSC (n = 10,172), the interval from collapse to ROSC at pre-hospital is 16 (10–25) min. Table 2 shows characteristics of outcomes in patients with the witnessed bystander CPR (the derivation and validation dataset combined). Neurologically favorable outcome at one month was achieved in 4,157/69,536 (6.0%) and one-month survival in 7,334/69,648 (10.4%).

**Table 1:** Characteristics of patients, bystander CPR, EMS, and the interval of CPR (The derivation and validation data set combined).

Patients with the witnessed bystander CPR (n = 69,648)		
Variables		Mean±SD or counts (percentages)
Age		74.6±15.8
Gender (Male)		40,288 (57.8)
Category of bystander	Family member	36,954 (53.0)
	Friends	2,844 (4.1)
	Colleagues	2,338 (3.4)
	Passersby	2,617 (3.8)
	Other laypersons	24,372 (35.0)
	Health care providers	463 (0.7)
	Others	60 (0.1)
Conventional CPR (vs. chest-compression only CPR)		27,950 (40.7)
AED by bystander		1,498 (2.2)
Dispatcher assisted with CPR		40,248 (57.9)
Initial rhythm of ECG	VF	11,374 (16.3)
	Pulseless VT	245 (0.4)
	PEA	21,144 (30.4)
	Asystole	33,406 (48.0)
	Others	3,479 (0.5)
DC by EMS		14,259 (20.6)
Kinds of defibrillator	Monophase	3,881 (5.8)
	Biphase	10,438 (15.7)
	No use	52,054 (78.4)
Category of airway tools by EMS	LM	6,038 (8.7)
	Esophageal obturator	21,615 (31.0)
	Intubation	5,593 (8.0)
	No use	36,402 (52.3)
IV by EMS		16,383 (23.7)
Epinephrine use by EMS		6,075 (8.8)
Cardiac cause		40,424 (58.0)
The interval from collapse to bystander CPR course (min, median (Q1–Q3))		
Interval from collapse to CPR initiation		1 (0–3)
Interval from collapse to ambulance arrival		9 (7–12)
Interval from collapse to hospital arrival		32 (26–41)
Interval from collapse to ROSC at pre-hospital (n = 10,172)		16 (10–25)

Missing data are; AED by bystander (1,608), Dispatcher assisted with CPR (162), Kinds of Defibrillator (3,275).

CPR = Cardiopulmonary resuscitation, SD = Standard deviation, AED = Automated external defibrillator, ECG = Electrocardiogram, VF = Ventricular fibrillation, VT = Ventricular tachycardia, PEA = Pulseless electrical activity, DC = Defibrillator cardioversion, EMS = Emergency medical service staff, LM = Lar y neal mask, IV = Intravenous fluid, ROSC = Return of spontaneous circulation, Q1 = 25% interquartile, Q3 = 75% interquartile.

**Table 2:** Characteristics of outcomes in patients with the witnessed bystander CPR (The derivation and validation data set combined).

Patients with the witnessed bystander CPR (n = 69,648)		
Outcome		Counts (Percentages)
One month survival		7,334 (10.5)
CPC	Favorable (CPC 1, 2)	4,157 (6.0)
	CPC 1	3,480 (5.0)
	CPC 2	677 (1.0)
	Poor (CPC 3, 4, 5)	65,379 (94.0)
	CPC 3	1,009 (1.5)
	CPC 4	2,178 (3.1)
	CPC 5	62,192 (89.4)

Missing data = CPC (112).

CPR = Cardiopulmonary resuscitation, CPC = Cerebral performance categories.

**Figure 3:**
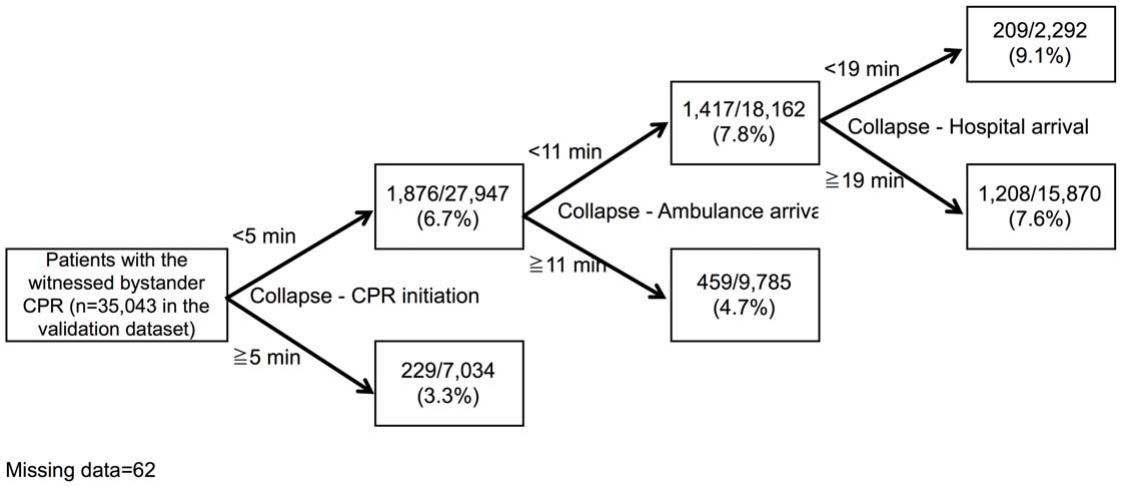
The partitioning model of the intervals of CPR for predicting the neurologically favorable outcome at one month in the validation dataset.

**Figure 4:**
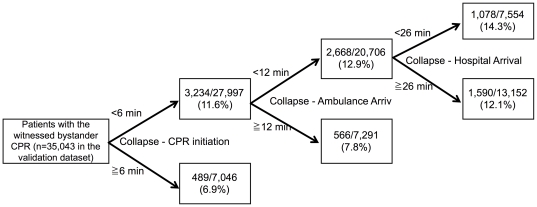
The partitioning model of the intervals of CPR for predicting the one month survival in the validation dataset.

The partitioning model for predicting the neurologically favorable outcome at one month using the derivation dataset showed 5 min for the optimal recommended threshold of the interval from collapse to CPR initiation, 11 min for the optimal recommended threshold of the interval from collapse to ambulance arrival, and 19 min for the optimal recommended threshold of the interval from collapse to hospital arrival (Figure 3). The partitioning model for predicting the one-month survival using the derivation dataset showed 6 min for the optimal recommended threshold of the interval from collapse to CPR initiation, 12 min for the optimal recommended threshold of the interval from collapse to ambulance arrival, and 26 min for the optimal recommended threshold of the interval from collapse to hospital arrival (Figure 4).

The partitioning model for predicting the neurologically favorable outcome at one month using the validation dataset produced the final node in which, the intervals were within the optimal recommended threshold of the intervals, with a significantly higher proportion of neurologically favorable outcome at one month (9.1%) compared to other nodes (Figure 3). Another partitioning model for predicting the one-month survival using the validation dataset produced the final node in which, the intervals were within optimal recommended threshold of the intervals, with a significantly higher proportion of one-month survival (14.3%) compared to other nodes (Figure 4).

**Figure 5:**
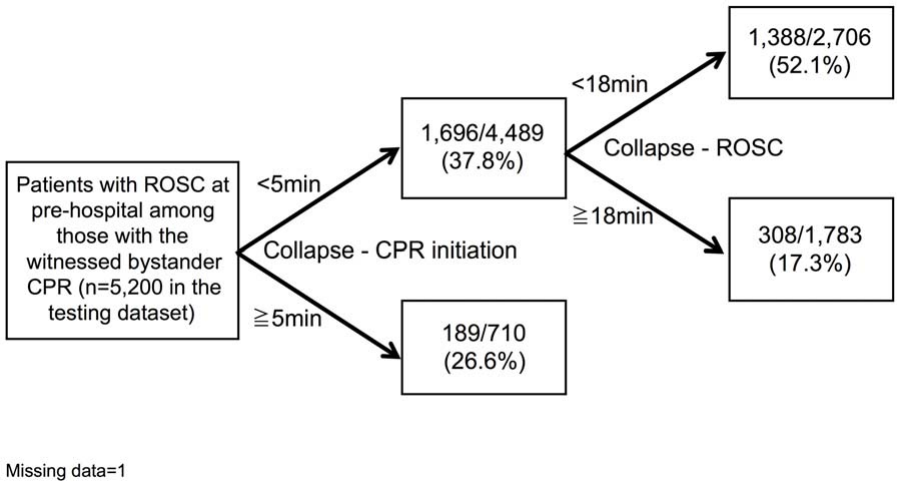
The subgroup partitioning model in those with pre-hospital ROSC for predicting the neurologically favorable outcome at one month using the testing dataset.

**Figure 6:**
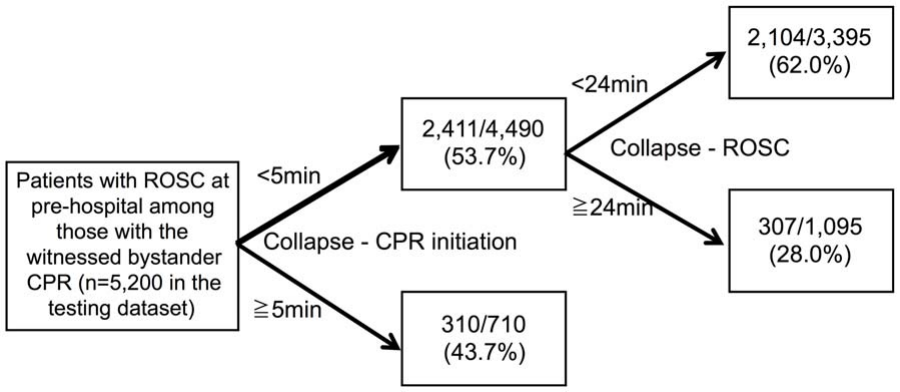
The subgroup partitioning model in those with pre-hospital ROSC for predicting the one month survival using the testing dataset.

Subgroup partitioning model in those with pre-hospital ROSC for predicting the neurologically favorable outcome at one month using the training dataset showed 5 min for the optimal recommended threshold of the interval from collapse to CPR initiation and 18 min for the optimal recommended threshold of the interval from collapse to ROSC (Figure 5). Subgroup partitioning model in those with pre-hospital ROSC for predicting one-month survival using the training dataset showed 5 min for the optimal recommended threshold of the interval from collapse to CPR initiation and 24 min for the optimal recommended threshold of the interval from collapse to ROSC (Figure 6).

The partitioning model for predicting the neurologically favorable outcome at one month using the testing dataset produced the final node in which, all intervals were within the optimal recommended threshold of the intervals, with a significantly higher proportion of neurologically favorable outcome at one month (52.1%) compared to other nodes (Figure 5). Another partitioning model for predicting the one-month survival using the testing dataset produced the final node in which, intervals were within the optimal recommended threshold of the intervals, with a significantly higher proportion of one-month survival (62.0%) compared to other nodes (Figure 6).

## Discussion

To our knowledge, this is the first study focusing on the optimal recommended intervals of CPR course for better clinical outcomes. For achieving neurologically favorable outcome at one month, we found the following results: bystander CPR should be initiated within 5 min from the time of collapse; the ambulances should arrive at the scene within 11 min after the time of collapse; the ambulances should bring patients to hospitals within 19 min after the time of collapse. One-minute delay from 5 min after collapse could be associated with loss of higher brain function. When patients with CPA received CPR within 5 min by witnessed bystanders and showed ROSC within 18 min, they had about 50% chance for achieving neurologically favorable outcome.

Clinically, it makes sense that starting CPR within 5 min is effective for achieving neurologically favorable outcome. The Seattle and Norway landmark studies have demonstrated that pre-arrest CPR is recommended for prolonged VF when emergency service providers arrive greater than 4 or 5 minutes although they focused on only patients with VF [14], [15]. Our research provides a time-based partitioning model for predicting neurologically favorable outcome in adult population with out-of-hospital CPA. Thus, our research adds to providing evidence-based information to their results. The SOS-Kanto study used 4 min as the threshold of the interval from collapse to CPR initiation for good outcome in adjusting the time interval as a predicting variable [16]. However, there was no appropriate statistical proof for this 4 min-cutoff in their study. This cutoff time point seemed to be used because the mean of the interval was 4 min, instead of 2.8 min in our study. Japanese studies showed the interval from a witnessed event to bystander CPR or CPR by EMS was significantly related to the outcomes of CPR. However, they did not determine the threshold of the intervals for obtaining good outcomes [10], [17]. Although it is clear that earlier CPR initiation is preferable, we demonstrated that 5 min is the threshold of CPR initiation for obtaining better outcomes. Thus, our results are novel in terms of showing the optimal recommended threshold of the interval from collapse to CPR initiation. It also convinces that even within one-minute delay of CPR is crucial for higher brain function as an animal study showed [4]. Traditional golden hour principle by M. Cara [2] also showed its importance.

Our results are compatible to recommendation for immediate chest compression after collapse by the 2010 AHA Guidelines for CPR and ECC [18]. By the revised sequence of chest compression-airway-breathing (C-A-B), chest compressions can be initiated sooner and ventilation could be only minimally delayed until completion of the first cycle of chest compressions because preceding two ventilations may need approximately 10 seconds. The simplified BLS algorithm in the 2010 guidelines also removed “Look, Listen and Feel” from the algorithm because performance of these steps is inconsistent and time consuming [18]. These revisions of the 2010 guidelines could save one min that was indicated in our study.

Our study indicated 11 min as the recommended threshold for interval from collapse to ambulance arrival at the scene. Because the mean interval of ambulance arrival in Japan in 2009 was 7 min 54 sec, this recommendation could be achieved for most patients [9]. However, if patients could not show ROSC at pre-hospital, their chances of favorable neurological outcomes would become low because the mean interval of hospital arrival in Japan in 2009 was 36 min 6 sec [9], although they might arrive to hospitals earlier in big cities. The previous study showed that the outcomes of CPA had regional variations [19]. Thus, the regional distance from hospitals may also be one of important factors to better outcomes among CPA patients.

About a half of patients with ROSC within 18 min had neurologically favorable outcome in comparison to 17.3% with over 18 min to ROSC at pre-hospital. Along with the results of our previous study [20], this result can be used for predicting better outcomes even at the time of hospital admission. Although results of these models should not be used to override careful clinical judgment in individual cases, these models can aid us to aim shorter interval time to CPR initiation and to predict outcomes among those with ROSC. We know it is better to start CPR as soon as possible. Thus, our results may be limited to determine protocols and recommendations. However, it is also true that there are several barriers, including hesitation and fear at clinical situation. Our results suggest that the barriers should be overcome and CPR should be started at least within 5 minutes. Moreover, despite regular updates of guidelines for the management of cardiac arrests, the rate of survival has been almost unchanged for more than 30 years [9]. Studies focused on time factors are needed more than changing methods of CPR in management of out-of hospital CPA.

There are several limitations. First, the time of collapse was determined through interviews with bystanders. It could be difficult to recall the exact time of onset of events in emergency situations. There might have been recall bias. However, these data were considered as relatively reliable because EMS checked the time of onset of events using the central time recordings at FDMA from time of ambulance call to arrival time to the scenes. Also, the EMS providers collecting the data had the same interview styles. While these criteria would exclude gross inaccuracies, the actual time used in the analysis was still bystander reported which could be also subject to recall bias. Because our model used the bystander reported time as the true number, the confidence intervals produced and utilized by the modeling software were narrower than reality. Second, the database might have included patients without CPA because it would have been difficult for laypersons to determine whether patients had CPA or not in emergency situations. Thus, our results might have overestimated the optimal intervals of CPR. However, our results are still useful because recommendation of these intervals could be stricter than the reality. Third, we did not analyze the interval from collapse to public AED use (or DC use by EMS) because of the small sample size. However, this variable could be potentially correlated with outcomes especially in CPA patients caused by cardiac arrhythmia. Forth, the guideline of resuscitation had been slightly changed during this study period. It might have influenced the interval from collapse to initiation of CPR. However, the patient outcomes provided by this change had improved because the number of laypersons who attempted CPR has been recently increased in Japan [9]. Fifth, this is prospective population-based observational study. However, prospective randomized control study may not be best way in urgent and complicated situations. Our results illustrate an important message as more likely a public health issue than resuscitation science [9]. Finally, this data was derived from only single country. Further external validations are needed.

In conclusion, CPR should be started as soon as possible, preferably within 5 minutes. One-minute delay after 5 min from the time of collapse could lead to loss of higher brain function. When patients with out-of-hospital CPA receive CPR within 5 min by witnessed bystanders and show ROSC within 18 min, they have about 50% chance to recover their neurological functions favorably.
